# Myometrial immune cells contribute to term parturition, preterm labour and post-partum involution in mice

**DOI:** 10.1111/j.1582-4934.2012.01650.x

**Published:** 2012-12-04

**Authors:** Oksana Shynlova, Tamara Nedd-Roderique, Yunqing Li, Anna Dorogin, Stephen J Lye

**Affiliations:** a Samuel Lunenfeld Research Institute, Mount Sinai HospitalToronto, Canada; b Department of Physiology, University of TorontoCanada; c Department of Obstetrics & Gynecology, University of TorontoCanada

**Keywords:** myometrium, leucocyte infiltration, cytokines, chemokines, infection, mifepristone

## Abstract

This study aimed to determine the mechanism of uterine activation during labour, both term (TL) and preterm (PTL). We hypothesized that the peripheral leucocytes are recruited to uterine tissues by locally produced cytokines where they contribute to the initiation of parturition. Mouse uteri were collected (*i*) during gestation, TL and post-partum (PP), (*ii*) during PTL initiated by intrauterine infusion of LPS (125 μg) or (*iii*) injection of the progesterone receptor antagonist RU486 and analysed for multiple cytokine expression levels by real-time polymerase chain reaction (RT-PCR) and 23-plex Cytokine assay or enzymatically dispersed for assessment of immune cell populations. Markers of myeloid cell differentiation (Gr1, Neu7/4 and F4/80) were evaluated by FACS to define tissue macrophages (Macs), monocytes (M) and neutrophils (N) and by immunohistochemistry to detect tissue Macs and N. Our results indicate that: (1) Macs were elevated in mouse myometrium before TL (*P* < 0.05) followed by an increase in M and N; these changes were accompanied by an increase in multiple pro-inflammatory cytokines/chemokines genes. The expression of corresponding proteins increased PP. (2) TL and RU486-PTL models showed similar gene/protein expression profiles, (3) LPS-PTL was characterized by strong pro-inflammatory response and massive influx of N in myometrial tissues showing a pattern different from TL and RU486-PTL, (4) The PP period appears similar in all three models, with elevated myometrial cytokine levels and high infiltration of immune cells. We concluded that leucocytes infiltrate myometrium around the time of parturition implicating their potential role in labour activation (both term and preterm) and major role in PP uterine involution.

## Introduction

Rates of preterm birth (PTB) have risen over the past two decades. Approximately half of PTB is of unknown etiology, whereas uterine infection, leading to chorioamnionitis and premature rupture of foetal membranes, is responsible for 30% of all PTB. Although a number of risk factors for PTB have been identified, prevention remains a great challenge as our understanding of the events preceding human parturition is still incomplete. Current therapies directed to inhibit myometrial contractile activity have not reduced the incidence of PTB [1, 2]. Their lack of effectiveness is likely related to the fact that they target a late stage in the labour cascade, when irreversible changes in reproductive tissues have already occurred. Understanding of the molecular and cellular events preceding the onset of labour both term and preterm is necessary for if we are to develop effective treatment to prevent PTB.

One factor that appears to be a common element of both infection and idiopathic PTB is the presence of an inflammatory state. Cervical ripening, an early event in normal labour, is characterized by an accumulation of leucocytes (predominantly macrophages and neutrophils) in the cervical stroma [3]. Many morphological studies have shown that the human myometrium is infiltrated by inflammatory cells during spontaneous non-complicated labour at term (TL) as well as those complicated by chorioamnionitis [4, 5]. We have similarly shown a large infiltration of macrophage into the myometrium and decidua at term in the pregnant rat model [6]. The decidual and foetal membranes exhibit a similar inflammatory pattern [4, 5]. Norman *et al*. hypothesized that the myometrium and foetal membranes play complementary roles during the process of labour–the trigger to parturition being delivered from foetal membranes (possibly through signals received from the foetus [7]) with subsequent leucocyte invasion stimulated within the myometrium to sustain and amplify the process of parturition [8, 9]. We suggest that within each uterine/intrauterine compartment immunoregulatory cytokines contribute to the induction of local inflammatory reactions (including cervical ripening, foetal membrane rupture, decidual activation and increased myometrial contractility) that together stimulate the process of labour. Inflammatory cells, which produce a variety of pro-inflammatory cytokines, reside and traffic within discrete regions of the pregnant uterus and account for 10% and 22% of cells in virgin and pregnant murine uteri respectively [10]. Immune cells appear to be attracted to the site of inflammation by chemokines (CCL2, CXCL1 and CXCL8). Leucocytes are a rich source of pro-inflammatory cytokines (IL1β, IL6 and TNF-α) [11, 12] and prostaglandins and hence are capable of initiating or amplifying an inflammatory cascade and triggering labour by activating the decidua and adjacent myometrium. Importantly, uterine tissues from preterm deliveries (with and without intrauterine infection) show a correlation between cytokine levels and the leucocyte infiltration, suggesting a direct link between the host response to infection and the onset of PTB [13, 14].

We hypothesize, therefore, that in preparation for labour peripheral leucocytes are recruited into myometrium by locally produced cytokines. Our study utilized well-characterized *in vivo* mouse models of normal pregnancy, spontaneous TL and post-partum, as well as non-infection/sterile PTL (a model for ‘idiopathic’ human PTL) induced by progesterone antagonist mifepristone (RU486) and infectious PTL induced by intrauterine infusion of Lipopolysaccharide (LPS; 125 μg) [15–17]. Using real-time polymerase chain reaction (RT-PCR), and 23-plex Luminex assays we investigated (1) the gene/protein expression profile of multiple cytokines in the mouse myometrium and (2) quantified the infiltration of leucocytes (monocytes, macrophages, neutrophils, eosinophils) in the myometrium in late gestation (GD15), during active parturition (both term and preterm) and early post-partum by multi-parameter flow cytometry (FACS) and stereological immunohistochemistry (IHC). Our data provide new understanding of the role of myometrial inflammation in labour initiation and in uterine tissue remodelling shortly after birth.

## Materials and methods

### Animal model

Hsd:ICR (CD-1) outbred mice used for these experiments were purchased from Harlan Laboratories (http://www.harlan.com/). All mice were housed under specific pathogen-free conditions at the Toronto Centre for Phenogenomics (TCP) on a 12L:12D cycle and were administered food and water *ad libitum*. All animal experiments were approved by the TCP animal care committee. Female mice were mated overnight with males and the day of vaginal plug detection was designated gestational day (GD) 0.5 of pregnancy. The average time of delivery was the early morning of GD 19. Our criteria for labour were based on delivery of at least one pup from average number of 14 in two uterine horns.

### Experimental design

#### Normal pregnancy and term labour

Animals were killed by carbon dioxide inhalation and myometrial samples were collected on GD 15.5, 18, term labour (TL) and 2–6 hrs post-partum (PP). Tissue was collected at 10 a.m. on all days with the exceptions of the labour sample (TL) that was collected once the animals had delivered at least one pup. The part of uterine horn close to cervix from where foetus was already expelled was removed and discarded; the remainder was collected for analysis. For each day of gestation, tissue was collected from 4 to 12 different animals.

#### LPS-induced preterm labour

The lipopolysaccharide (LPS) used for this study was isolated from *E. coli*, serotype 055:B5 (Sigma-Aldrich, St Louis, MO, USA). On GD 15.5, mice underwent mini-laparotomy under general anaesthesia (isoflurane) with intrauterine infusion of 125 μg LPS in 100 μl of sterile saline between two lower amniotic sacs (LPS group) or intrauterine infusion of 100 μl sterile saline (Sham group). Animals (*n* = 4–8 per group) were killed during LPS-induced PTL or 24 hrs after sham surgery. Post-partum samples were collected 2–6 hrs after preterm delivery (LPS PP group).

#### RU486-induced preterm labour

On GD 15.5 of gestation, two groups of mice were injected subcutaneously with either RU486 (150 μg in 100 μl corn oil containing 10% EtOH, 17β-hydroxy-11β-[4-dimethylaminophenyl]-17-[1-propynyl]-estra-4,10-dien-3-ne; Mifepristone; Biomol International, Plymouth, PA, USA) or vehicle. Myometrial samples were collected from RU486-treated animals after delivery of at least one pup (RU486 group), or 24 hrs after injection of the corn oil/ethanol solvent in control mice (Vehicle group) (*n* = 4–8/group). Post-partum samples were collected 2–6 hrs after preterm delivery (RU486 PP group).

### Tissue collection for cytokine expression analysis and immunohistochemistry

For RNA and protein extraction, one uterine horn was placed into ice-cold PBS, bisected longitudinally and dissected away from both pups and placentas. The decidua basalis was cut away from the myometrial tissue, the decidua parietalis were carefully removed from the myometrial tissue by mechanical scraping on ice. This removed the entire luminal and glandular epithelium and the majority of the uterine stroma. The myometrial tissue was flash-frozen in liquid nitrogen and stored at −80°C. The second uterine horn was collected for immunohistochemical analysis: the whole intact uterine horn was cut into 5–10 mm segments and placed in 10% neutral-buffered formalin (Harleco, Baltimore, MD, USA) or 4% paraformaldehyde (PFA, Electron Microscopy Sciences, Hartfield, PA, USA) for fixation. Samples were fixed for 24 hrs at 4°C.

### RT-PCR analysis

Total RNA was extracted from the frozen mouse myometrium using TRIZOL (Gibco BRL, Burlington, ON, Canada) according to manufacturer's instructions. RNA samples were column purified using RNeasy Mini Kit (Qiagen, Mississauga, ON, Canada), and treated with DNase I (Qiagen) to remove genomic DNA contamination. The process was quality controlled by measuring yield (μg), concentration (μg/l) and 260:280 ratios *via* spectrometry using Nanodrop ND-1000 (Thermo Scientific Inc., Mississauga, ON, Canada) and sample integrity using Experion system (Bio-Rad, Mississauga, ON, Canada). cDNA synthesis was performed as per manufacturer's protocols (iScript cDNA synthesis kit, Bio-Rad). Quantitative RT-PCR was performed with Luminoct SYBR Green QPCR READYMIX (Sigma-Aldrich), CFX-96 real time system C1000 thermal cycler (Bio-Rad) and a specific pairs of primers (see Table 1). Aliquots (10 ng) of cDNA were used for each PCR reaction run in triplicates. A cycle threshold (Ct) value was recorded for each sample. Each gene was normalized to the expression of three housekeeping genes (*Ppia*, *Tbp*, *Hprt*) by CFX Manager (version 2.1) software and relative expression was calculated for mouse normal gestation using the average of GD15 as the external calibrator in the comparative Ct method (see ABI user bulletin no. 2). Gene expression for LPS- and RU486-treated animals was presented as the average fold change relative to the Sham or Vehicle, except for *Pghs2, Otr* and *Gja1* where the average of GD15 was used as an external calibrator.

**Table 1:** Real-time PCR primer sequences of a panel of genes involved in inflammatory response and housekeeping genes

Target genes	Primer sequences	GenBank accession #
*Il1a*	Forward 5′-GTGTTGCTGAAGGAGTTGCC-3′ Reverse 5′-CTGGATAAGCAGCTGATGTG-3′	NM_010554
*Il1b*	Forward 5′-GGACCCCAAAAGATGAAGGGCTGC-3′ Reverse 5′-GCTCTTGTTGATGTGCTGCTGCG-3′	NM_008361
*Il-6*	Forward 5′-CCTCTCTGCAAGAGACTTCC-3′ Reverse 5′-CTCCGGACTTGTGAAGTAGG-3′	NM_031168
*Il-12b*	Forward 5′-AACCAGAAAGGTGCGTTCCTC-3′ Reverse 5′-ATGCCCACTTGCTGCATGA-3′	NM_008352
TNF-*α*	Forward 5′-ATGGCCCAGACCCTCACACTCA-3′ Reverse 5′-TGGTGGTTTGCTACGACGTGGG-3′	NM_013693
*Csf2*	Forward 5′-TCGAGCAGGGTCTACGGGGC-3′ Reverse 5′-GTCCGTTTCCGGAGTTGGGGG-3′	NM_009969
*Ccl2*	Forward 5′-AGGTGTCCCAAAGAAGCTGTA-3′ Reverse5′-TCTGGACCCATTCCTTCTTG-3′	NM_011333
*Cxcl1*	Forward 5′-CCTGCAGACCATGGCTGGGAT-3′ Reverse 5′-GTGTGGCTATGACTTCGGTTTGGG-3′	NM_008176
*Ccl3*	Forward 5′-AGCTGACACCCCGACTGCCT-3′ Reverse 5′-TCAGGAAAATGACACCTGGCTGGGA-3′	NM_011337
*Ccl4*	Forward 5′-AGCCAGCTGTGGTATTCCTGACCA-3′ Reverse 5′-TCATGTACTCAGTGACCCAGGGCT-3′	NM_013652
*Cxcl2*	Forward 5′-GTTTGCCTTGACCCTGAAGCCCC-3′ Reverse 5′-CCAGGTCAGTTAGCCTTGCCTTTGT-3′	NM_009140
*Gja1*	Forward 5′-GGTCTGAGAGCCCGAACTCTCCT-3′ Reverse 5′-ACCCATGTCTGGGCACCTCTCTT-3′	NM_010288
*Oxtr*	Forward 5′-TCATCGTGTGCTGGACGCCT-3′ Reverse 5′-TGTTGAGGCTGGCCAAGAGCAT-3′	NM_001081147
*Hpgd*	Forward 5′-TCGGATTCACACGCTCAGCAGC-3′ Reverse 5′-TGTGTCCACAAAGCCTGGGCAA-3′	NM_008278
*Ptgs2*	Forward 5′-TGCCCAGCACTTCACCCATCA-3′ Reverse 5′-AGTCCACTCCATGGCCCAGTCC-3′	NM_011198.3
*Il-10*	Forward 5′-GCGGCTGAGGCGCTGTCAT-3′ Reverse 5′-GGCCTTGTAGACACCTTGGTCTTGG-3′	NM_010548
*Hprt*	Forward 5′-CAGTCCCAGCGTCGTGAT-3′ Reverse 5′-CAAGTCTTTCAGTCCTGTCCATAA-3′	NM_013556.2
*Ppia*	Forward 5′-CACCGTGTTCTTCGACATCA-3′ Reverse 5′-CCAGTGCTCAGAGCTCGAAAG-3′	NM_008907.1
*Tbp*	Forward 5′-TCCCAAGCGATTTGCTGCAGTCATC-3′ Reverse 5′-ACTCTTGGCTCCTGTGCACACCA-3′	NM_013684

### Luminex assay

Frozen myometrial tissue samples were crushed under liquid nitrogen and homogenized in bicine lysis buffer [25 mM Bicine, 150 mM NaCl, pH 7.6] supplemented with 100 μM sodium orthovanadate and protease inhibitor cocktail tablets (CompleteTM Mini, Roche, Quebec, Canada). Samples were spun at 12,000 g for 15 min. at 4°C, the supernatant was transferred to a fresh tube to obtain a crude protein lysate and stored at −20°C until assayed. Total protein concentrations were determined using Bio-Rad assay (Bio-Rad). The optimal sample protein content for the measurement of multiple immunoreactive cytokines was established by serial dilution. Two hundred and fifty micrograms of protein from tissue homogenates of each myometrial sample were used for multiplex assay. Tissue cytokine levels were quantified using Bio-plex Pro mouse Cytokine 23-plex array kit (Bio-Rad). Multiplex assay was performed on Luminex 200 system and Bioplex HTF (Bio-Rad) in accordance with the manufacturer's instructions. Standards and each sample were analysed in duplicate. Data analysis was performed using Bio-Plex Manager, version 5.0 (Bio-Rad) and presented as concentrations (pg/ml).

### Tissue dispersion for flow cytometry

Uteri were dissected, the myometrium was separated from the decidua, embryos and yolk sacs were discarded. Myometria were finely minced with scissors and enzymatically dissociated in HBSS buffer with 10% FBS, containing Collagenase I (1 mg/ml; Sigma-Aldrich), DNAse (0.15 mg/ml; Roche) and Hyaluronidase, type 1S (50 U/ml; Sigma-Aldrich) for 2 hrs at 37°C with agitation. The tissues were manually pipetted every 30 min. to improve dispersion (repeated four times) prior to filtration. After incubation, the single-cell suspensions were passed three times through a syringe with an 18-gauge needle, followed by three passes through a 23-gauge needle, filtered through a 40-μm cell strainer and pelleted by centrifugation (500 × **g**, for 5 min. at 4°C).

### FACS analysis

Staining procedure was performed as described earlier [18] with some modifications. Briefly, dispersed cells were pre-treated with FcγR-blocking mAb 2.4G2 (BD Biosciences, San Diego, CA, USA) together with a combination of up to six directly conjugated fluorescent Abs and fixable dead cell green stain kit (Invitrogen, Eugene, OR, USA). Cells were incubated for 60 min. in the dark on ice. RBC lysis and cellular fixation was completed using BD FACS lysing solution (BD Biosciences). Cells were washed and samples were run within 24 hrs on a 2-laser 7-colour BD Biosciences FACS Aria flow cytometer using BD FACS Diva software (BD Biosciences). After the initial gating on forward-*versus*-side scatter plots, uterine cell populations were gated on all viable leucocytes and APC-Cy7-conjugated anti-CD45 antibody (clone30-F11, BD). The cells were then delineated into subsets with antibodies against F4/80 (BM8, C1:A3-1), neutrophils (Neu7/4), Ly6C/G (Gr-1, RB6-8C5) and Siglec F purchased from BD Biosciences and AbD Serotec and variously conjugated to FITC, RPE-Alexa Fluor 647, PerCP-Cy5.5, PE, Alexa Fluor 647, PE-Cy7. Isotype controls were used to assess background fluorescence; FMO controls were used to verify subset gates.

### Immunohistochemistry

The formalin-fixed uterine tissues were gradually dehydrated in ethanol and embedded in paraffin. Sections of 5 μm thickness were collected on superfrost plus slides (Fisher Scientific, Nepean, ON, Canada). Paraffin sections were deparaffinized and rehydrated. After immersion in 3% hydrogen peroxide (Fisher Scientific, Fair Lawn, NJ, USA), the antigens were unmasked using a microwave heating retrieval treatment in 10 mM sodium citrate solution (pH6) for formalin-fixed tissue and using 0.125% trypsin for PFA-fixed tissue. Blocking was performed for 1 hr with DAKO protein serum-free blocking solution (DAKO Corporation, Carpinteria, CA, USA). Formalin-fixed tissue was incubated with primary anti-Neu7/4 monoclonal rat antibody (1:100; Cedarlane, Burlington, ON, Canada) overnight. Neu7/4 recognizes the polymorphic 40 kD antigen expressed by polymorphonuclear cells, but absent on resident tissue macrophages. Neu7/4 has low expression on monocytes. PFA-fixed tissue was incubated with primary anti-F4/80 monoclonal rat antibody (1:100; BioLegend, San Diego, CA, USA). F4/80 recognizes the 160 kD glycoprotein expressed majorly by murine macrophages and has low expression on monocytes and eosinophils. For the negative controls, ChromPure non-specific rat IgGs (Jackson ImmunoResearch Laboratories, West Grove, PA, USA) were used at the same concentration and sections were also incubated with secondary antibodies in the absence of primary antibodies. Detection was accomplished using biotinylated rabbit anti-rat IgG (1:200; Vector Laboratories, Burlingame, CA, USA) in combination with Streptavidin HRP (DAKO Corporation). Final visualization was achieved using Dako liquid DAB + substrate chromogen system (DAKO Corporation). Counterstaining with Harris' Hematoxylin (Sigma-Aldrich) was carried out before slides were mounted with Cytoseal XYL (Thermo Scientific, Kalamazoo, MI, USA).

### Assessment of leucocyte infiltration using NewCast software

Infiltration of macrophages and neutrophils was quantified using NewCast stereology software with systematic randomized sampling of 1–2% of the total myometrial area. Uterine tissues from different gestational days, TL, from LPS PTL and RU486 PTL groups along with appropriate controls were observed on an Olympus BX61 (Olympus America Inc., Center Valley, PA, USA) microscope and recorded using an Olympus DP72 camera (Olympus). The population of leucocytes was assessed for Neu7/4 and F4/80 immunostaining to identify tissue neutrophils/monocytes and macrophages/monocytes respectively. In each uterine tissue sample NewCast software, part of Visiopharm integrator system 3.6.5.0, was used to generate 25–45 non-contiguous, randomly selected fields of myometrium. Cells positive for Neu7/4 and F4/80 and total nuclei in each field were counted at 20× magnification. The number of cells having positive staining was divided by total number of cells in the field and multiplied by 100 to determine percentages of Neu7/4 or F4/80 positive cells.

### Statistical analysis

Grubbs outlier test was utilized to identify and exclude outliers from all data sets. To determine differences between groups, we subjected gestational profiles, LPS and RU486 profiles to a one-way analysis of variance (anova) followed by Newman-Keuls post-test (for normally distributed data) or to Kruskal-Wallis non-parametric test followed by Dunns post-test (for not normally distributed data). Normality test and equal variance test were performed with Sigma Stat statistical program and where required, the data were transformed by an appropriate method (usually natural logarithm function) to obtain a normal distribution. The choice as to whether anova or Kruskal-Wallis test was used is based upon the results of the test for statistical normality. The rank methods, Kruskal-Wallis test, were used when the data and the transformed data both reject the hypothesis of normality. Statistical analysis was carried out using GraphPad Prism (version 4; GraphPad Software Inc., La Jolla, CA, USA) for cytokine expression data or Sigma Stat (version 3.11) for the leucocyte infiltration data with the level of significance set at *P* < 0.05.

## Results

### Myometrial cytokine expression at late gestation, term labour and post-partum

**Fig. 1:**
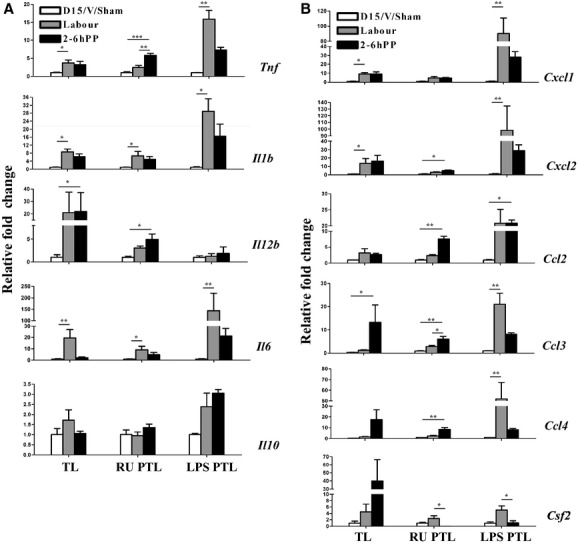
Changes in cytokine mRNA levels in the mouse myometrium during normal gestation, term labour and post-partum (TL group), LPS-induced PTL and post-partum (LPS PTL group) and RU486-induced PTL and post-partum (RU PTL group). (**A**) Pro-inflammatory (*Il1b*, *Il6*, *Il12b*, TNF) and anti-inflammatory (*Il10*) cytokines (**B**) Chemokines *Ccl2* (*Mcp1*), *Ccl3* (*Mip1a*), *Ccl4* (*Mip1b*), *Csf2* (*Gmscf*), *Cxcl1* (KC or Groa) and *Cxcl2* (*Mip2a*) gene expression were detected by real-time PCR (RT-PCR). Shown are non-labouring samples (GD15, RU Vehicle or LPS Sham, white bars), term and preterm labouring samples (grey bars) or myometrium samples collected 2–6 hrs post-partum (black bars). Results were expressed as an average ± SEM (*n* = 4–5). One-way anova was utilized followed by Newman-Keuls post-test (for *Il10*, *Ccl2*) and Kruskal-Wallis non-parametric test followed by Dunns post-test (for all other cytokines). Significant difference with GD15/RU486 Vehicle/Sham LPS is indicated by *(*P* < 0.05), **(*P* < 0.01) and ***(*P* < 0.001).

**Fig. 2:**
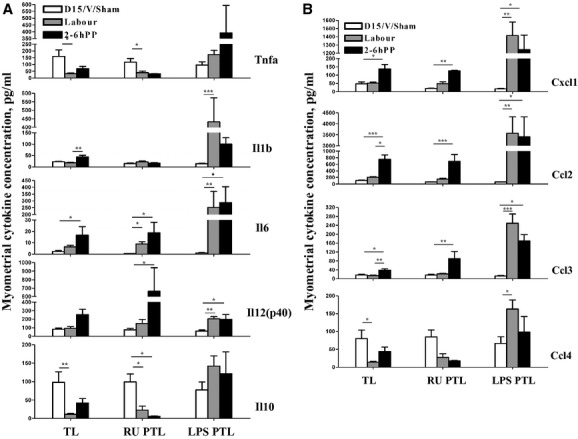
Changes in cytokine protein levels in the mouse myometrium during normal gestation, term labour and post-partum (TL group), LPS-induced PTL and post-partum (LPS PTL group) and RU486-induced PTL and post-partum (RU PTL group). (**A**) Pro-inflammatory (Il1b, Il6, Il12(p40), TNF-α) and anti-inflammatory (Il10) cytokines (**B**) Chemokines Ccl2 (Mcp1), Ccl3 (Mip1a), Ccl4 (Mip1b) and Cxcl1 (KC or Groa) protein expression were detected by multiplex magnetic bead assay. Shown are non-labouring samples (GD15, RU Vehicle or LPS Sham, white bars, *n* = 8 for all groups), term (*n* = 10) and preterm (*n* = 8) labouring samples (grey bars) or myometrium samples collected 2–6 hrs post-partum (*n* = 8, black bars). One-way anova was utilized followed by Newman-Keuls post-test for all cytokines in TL group except for Il6. Results were expressed as mean ± SEM. Kruskal-Wallis non-parametric test was utilized for cytokine analysis in LPS PTL and RU PTL groups followed by Dunns post-test. Results were expressed as an average ± SEM. Significant difference with GD15/RU486 Vehicle/Sham LPS is indicated by *(*P* < 0.05), **(*P* < 0.01) and ***(*P* < 0.001).

The expressions of multiple pro- and anti-inflammatory genes were evaluated by RT-PCR during late gestation (GD15), TL and PP. ‘Early response’ cytokines, tumour necrosis factor-α (TNF) and interleukin (Il)1β (*Il1b*), as well as *Il6* and *Il12* were significantly up-regulated during TL in parallel with the neutrophil chemoattractants (C-X-C motif) ligand1 (*Cxcl1*, also known as Neutrophil-activating protein 3 (NAP-3) or KC) and *Cxcl2* (also known as macrophage inflammatory protein 2-α, Mip2 α) (Fig. 1A). Monocyte chemoattractant *Ccl4* (also known as Macrophage inflammatory protein 1 β, Mip1β), granulocyte recruiter/activator *Ccl3* (also known as Macrophage inflammatory protein 1 α, Mip1α) and neutrophil/monocyte differentiating factor *Csf2* (granulocyte-macrophage colony-stimulating factor) transcript levels increased in PP myometrium 2–6 hrs after parturition. *Ccl2* transcript levels showed similar changes that were not significant. Anti-inflammatory cytokine *Il10* gene expression was not influenced at term parturition. The expression of corresponding proteins increased PP and thus temporally followed changes in cytokine transcripts. Using 23-plex protein assay, we detected a significant increase in Il1b, Il6 and Il12p40 pro-inflammatory cytokines, Cxcl1, Ccl2, Ccl3 chemokines and Csf3 (granulocyte colony-stimulating factor) in the mouse myometrium during the PP period (*P* < 0.05; Fig. 2A and B, Figure S1C). Surprisingly, protein levels of Ccl4, Il1a and Il10 were significantly decreased during labour and PP (*P* < 0.05). Other cytokines (*i.e*. TNF-α, Il3, Il4, Il12p70, Il17 and Ccl5) also displayed decreases in their protein expression during TL, whereas no change in the level of Il5, Il9, Il13, Infg, eotaxin and Csf2 was observed (see Figure S1A and B). Data in Figure 1A and B are summarized in Table S1, data in Figure 2A and B are summarized in Table S2.

### Myometrial cytokine expression during preterm labour

PTL was induced (1) by artificial progesterone blockade using mifepristone (non-infectious, sterile PTB [16, 17]) or (2) by intrauterine injection of 125 μg LPS (the well-known model of infectious PTB [15]) on GD 15 pregnant mice. Both treatments resulted in PTL within 24 hrs with no maternal mortality. The expression of multiple cytokine and chemokine genes was studied in myometrial samples collected during both models of PTL and 2–6 hrs PP. We noticed a similarity between cytokine profile discovered in myometrium collected from term labouring mice and RU486-induced PTL. Parallel to TL model, *Il1b* and *Il6* mRNA levels were significantly up-regulated during non-infectious RU486-induced PTL. Importantly *Il12*, TNF*-*α, *Ccl2*, *Ccl3*, *Ccl4* and *Cxcl2* transcripts were significantly elevated PP as compared to Vehicle and PTL (Fig. 1A and B and Table S1). In accordance with transcript levels, a significant increase in protein levels of pro-inflammatory cytokine Il6 and Il12p40 was detected by Bioplex assay in PP myometrium after RU486-induced PTL (*P* < 0.05, Fig. 2A and Table S2). Similar to TL, there was a significant increase in Ccl2, Ccl3 and Cxcl1 protein expression levels during PP period compared to Vehicle sample and PTL (*P* < 0.05; Fig. 2B). There was no change in Il1b, Il9, Csf1, IFNγ and Ccl5 (Fig. 2A, Figure S1 and Table S2), however, anti-inflammatory cytokine Il10 and pro-inflammatory cytokines Il1a, Il3, Il17, Il12p70 and TNF-α were significantly down-regulated during RU486-induced PTL and PP (*P* < 0.05; Fig. 2A and Figure S1). In addition Il5, Il13 and eotaxin protein levels were increased significantly PP (*P* < 0.05; Figure S1 and Table S2).

In contrast with TL and RU486-induced PTL, the gene and protein profile of LPS-induced PTL in mice shows a robust induction of the majority of pro-inflammatory cytokines and chemokines we studied. *Il1b*, *Il6*, TNF, *Il10*, *Ccl2*, *Ccl3*, *Ccl4*, *Cxcl1*, *Cxcl2*, *Csf2* myometrial transcript levels were up-regulated during LPS-induced PTL (*P* < 0.05; Fig. 1A and B and Table S1). In agreement with transcript levels, Il1b, Il6, Il12p40, as well as Il5, Il9, Il13, Il17, Ifng protein concentrations were significantly increased in the mouse myometrium during preterm parturition compared to Sham samples and for most of cytokines remained or further increased PP (*P* < 0.05; Fig. 2A, Figure S1 and Table S1). In addition, protein expression of all chemokines (Ccl2, Ccl3, Ccl4, Ccl5, Cxcl1, Cxcl2, Cxcl3 and eotaxin) was significantly higher in the myometrium during LPS-induced PTL compared to Sham and remained elevated PP (*P* < 0.05; Fig. 2B and Figure S1). There was no change in anti-inflammatory Il4 and Il10 proteins during LPS-induced PTL or PP compared to Sham.

### Myometrial CAP gene expression during preterm labour

**Fig. 3:**
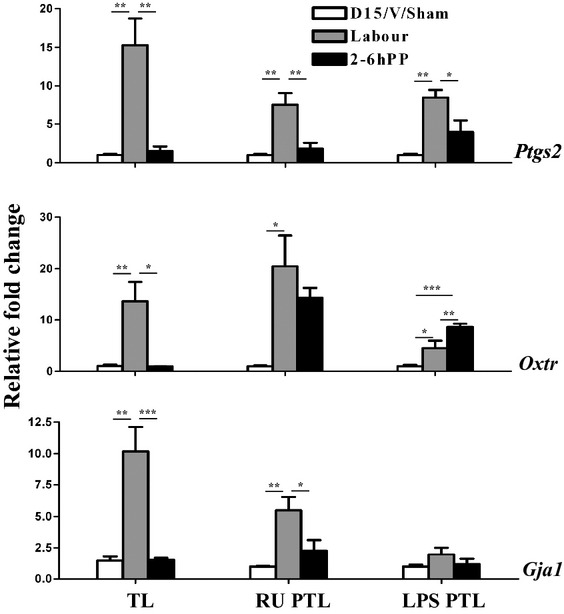
Genes up-regulated during term labour (TL) are induced by RU486 treatment (RU PTL) and intrauterine infusion of LPS (LPS PTL). Connexin 43 (*Gja1*), oxytocin receptor (*Oxtr*) and prostaglandin synthase 2 (*Ptgs2*, formerly Cox2) expression were assessed by RT-PCR in mouse myometrium. Shown are non-labouring samples (GD15, RU Vehicle or LPS Sham, white bars), term and preterm labouring samples (grey bars) or myometrium samples collected 2–6 hrs post-partum (black bars). One-way anova was utilized followed by Newman-Keuls post-test. Significant difference with GD15/RU486 Vehicle/Sham LPS is indicated by *(*P* < 0.05), **(*P* < 0.01) and ***(*P* < 0.001). Data represent mean ± SEM of four myometrium samples.

Genes known to be induced during TL [19, 20] were assessed by RT-PCR in PTL mouse myometrium. We attempted to determine whether changes in pro-inflammatory cytokines detected during PTL were associated with changes in the mRNA expression of well-known contraction-associated proteins *Gja1* (formerly connexin43), *Oxtr* (oxytocin receptor) and prostaglandin-cyclooxygenase-endoperoxide synthase 2 (*Ptgs2*, formerly Cox2), regulating prostaglandin synthesis [21]. Similar to TL, RU486 treatment significantly increases the expression of *Gja1*, *Oxtr* and *Ptgs2* transcripts during PTL (Fig. 3). The myometrial expression of all three genes decreased immediately after TL to the levels detected on GD15. In the LPS-treated mice, *Oxtr* and *Ptgs2* expression increased significantly during infectious PTL, but *Gja1* was not altered. In RU486-treated group *Gja1* and *Ptgs2* decreased after PTL, however, *Oxtr* transcript levels remained elevated during early PP in both model of PTL.

### Leucocyte infiltration throughout late gestation, term labour and post-partum in the mouse myometrium

**Fig. 4:**
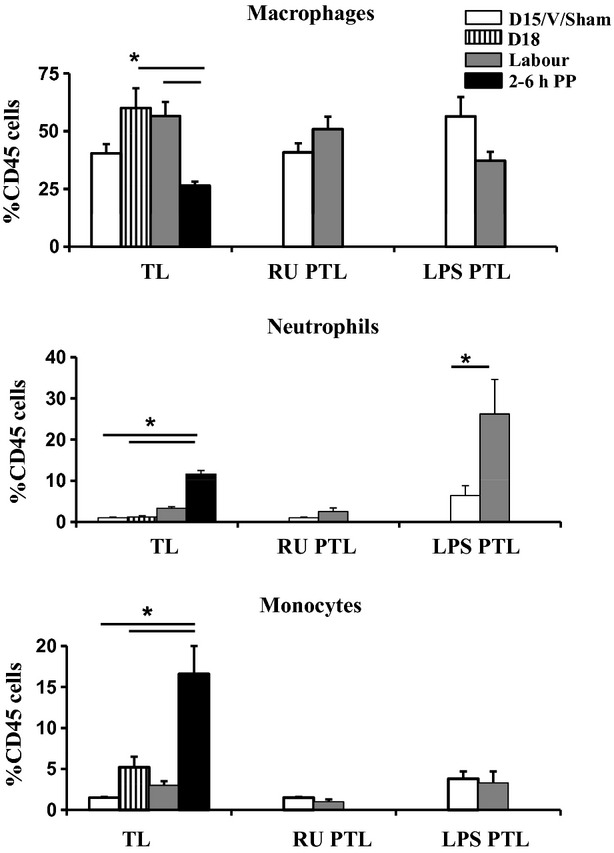
Recruitment of myeloid cells in the mouse myometrium. Myometrial suspensions were stained with anti-CD45, F4/80, Gr1 and Neu7/4. Leucocytes were gated on CD45 and further analysed on the Neu7/4 *versus* Gr1 dot plot. Macrophages were defined as F4/80^++^ and Neu7/4^−^. Monocytes were identified as Neu 7/4^++^ and Gr1^**−**^ to Gr1^**+/−**^. Neutrophils were defined as Neu7/4^+^, Gr1^++^ [18]. Myometrium was analysed during normal gestation, term labour and post-partum (TL), LPS-induced PTL (LPS PTL) and RU486-induced PTL (RU PTL). Shown are non-labouring samples (GD15, LPS Sham surgery or RU Vehicle, white bars, *n* = 6–8), GD18 (term not in labour, striped bars, *n* = 6), term labouring (*n* = 7) and preterm labouring samples (grey bars, *n* = 4) or post-partum samples (*n* = 6 black bars). Data represent mean ± SEM. Significant difference from GD15/RU486 Vehicle/Sham LPS is indicated by asterisk (*, *P* < 0.05).

To test the hypothesis that specific leucocyte populations are recruited to the myometrium in association with the onset of labour, infiltration of immune myeloid cells into the normal pregnant mouse myometrium was analysed by flow cytometry throughout different phases of pregnancy: late gestation (GD15 and GD18), during active TL and early PP (Fig. 4). Myometrial cell suspensions were stained with anti-CD45, anti-F4/80, anti-Siglec-F, anti-Gr1 and anti-Neu7/4 as described in [18]. Live leucocytes were gated on CD45 and further analysed on the Neu7/4 *versus* F4/80 dot plot (Figure S2). Neu7/4^++^ cells were further analysed on the Neu7/4 *versus* Gr1 plot. Monocytes were identified as Neu7/4^++^ and Gr1^low^ to Gr1^+/−^ cells. Neutrophils were defined as Neu7/4^+^, Gr1^++^ population. Macrophages were defined as F4/80^++^, Neu7/4^low^ cells. Population of cells defined as F4/80^+^, Neu7/4^low^ Gr1 strongly expressed the eosinophil-specific marker, Siglec-F [18].

**Fig. 5:**
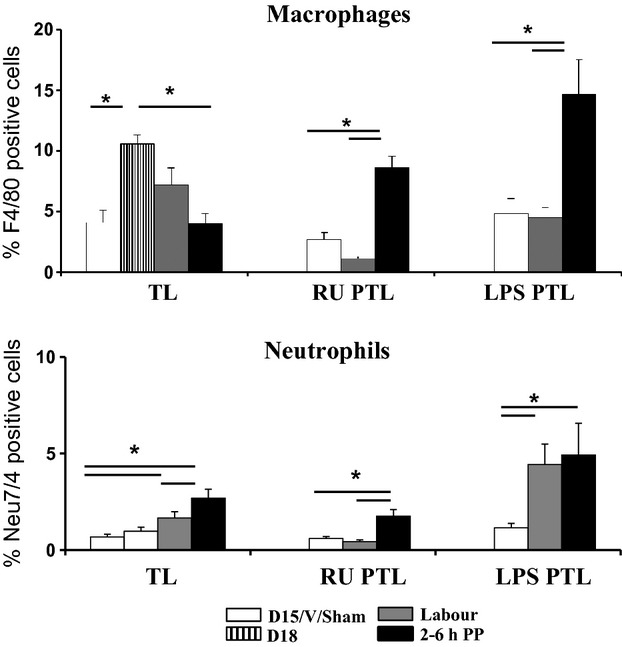
Neutrophil and macrophage infiltration into the mouse myometrium during TL and PTL. Neutrophils were identified using anti-Neu7/4 antibody and macrophages were identified using anti-F4/80 antibody. NewCast software was used to quantify neutrophil and macrophage numbers relative to total cell numbers in the myometrium during normal gestation, term labour and post-partum (TL), LPS-induced PTL and post-partum (LPS PTL) and RU486-induced PTL and post-partum (RU PTL). Shown are non-labouring samples (GD15, LPS Sham or RU Vehicle, white bars), GD18 (term not in labour, striped bars), term and preterm labouring samples (grey bars) or post-partum samples (black bars). Results were expressed as mean ± SEM (*n* = 4–12). Neutrophil infiltration data from the LPS PTL mouse model was transformed using the natural logarithm (ln) function to obtain a normal distribution. One-way anova was utilized followed by Newman-Keuls post-test. Significant difference with GD15/RU486 Vehicle/Sham LPS is indicated by *(*P* < 0.05).

Macrophages were the largest population of immune cells present in pregnant mouse myometrium, representing 40.8% of leucocytes (Fig. 4). They increased in number by GD18 (term) as compared to GD15, were elevated during spontaneous TL, however, by 2–6 hrs PP macrophage numbers in myometrium decreased back to levels observed during late gestation (*P* < 0.05; Figs 4 and 5). The number of myometrial monocytes (Neu7/4^++^, Gr1), but not neutrophils, increased at term (GD18) as compared to GD15 (1.5% *versus* 5.2%) and were dramatically up-regulated through the early PP period (16.6%, *P* < 0.01; Fig. 4, Figure S2A). Neutrophils (Neu7/4^+^, Gr1) were present in myometrium throughout late gestation, however, they did not increase until TL (1% *versus* 3.3%) and were significantly up-regulated PP (11.6%, *P* < 0.01; Fig. 4). Relative numbers of infiltrated myometrial monocytes and neutrophils during TL were much lower than macrophage numbers on all stages of pregnancy and labour. Tissue eosinophils were low (around 1%) and unchanged during gestation and early PP (Figure S2A).

NewCast software was used to correlate the flow cytometry data by quantifying neutrophils and macrophages immunostained *in situ*. Similar to FACS, immunohistochemical analysis indicate that myometrial macrophages (4.1% of all cells in pregnant uterine muscle, defined as F4/80 positive) were significantly increased at term (GD 18) as compared to late gestation (GD 15), and decreased back to levels observed during late gestation by PP (*P* < 0.05, Fig. 5 and Figure S3A) providing a direct evidence of immune cells extravasation into the tissue. The number of neutrophils, defined as cells stained positively for Neu7/4, were low during late gestation and TL, but significantly increased in myometrium during PP period (*P* < 0.05; Fig. 5 and Figure S3B). Importantly, in term mouse myometrium and during spontaneous TL, there was 7–10 times more macrophages than neutrophils. We cannot exclude the possibility that some of these positive cells were actually monocytes as they can faintly express both markers. It was previously reported that Neu7/4 can recognize monocytes along with neutrophils and that F4/80 has low expression on monocytes [22].

### Myometrial leucocyte infiltration during preterm labour

Flow cytometry was used to compare the inflammatory cell populations in two PTL models *versus* TL (Fig. 4, Figure S2B). Analysis of myometrial cell suspensions from LPS-induced preterm labouring mice showed a dramatic increase in tissue neutrophils as compare to the myometrium from the Sham group (6.4 *versus* 26.2%, *P* < 0.05). During RU486-induced PTL, the number of macrophages was unchanged and no infiltration of myeloid immune cells (N and M) into the myometrium was detected. No significant change in eosinophils was observed with any of the treatments (Figure S2B).

Recruitment of macrophages and neutrophils into the myometrium was also assessed *in situ* in both models of PTL. In accordance to flow cytometry results, no infiltration in immunopositive macrophages or neutrophils was detected in the myometrium during RU486-induced PTL (Fig. 5, Figure S3C). In contrast with sterile RU486-induced PTL, number of neutrophils was significantly increased in myometrium during infectious LPS-induced PTL as compared to the Sham group which directly correlates with our FACS data. Importantly, the number of neutrophils and macrophages increased significantly in the PP myometrium following both LPS and RU486-induced PTL (*P* < 0.05; Fig. 5, Figure S3D). During the PP period, three to five times more macrophages than neutrophils infiltrated in the mouse myometrium.

## Discussion

We previously demonstrated that there is a gradual activation and priming of the rat maternal immune system throughout pregnancy as compared to the non-pregnant state. A significant increase in the expression of Ccl2 gene and protein (also known as monocyte chemotactic protein-1, Mcp-1) was observed in the rat myometrium before and during the onset of TL *versus* non-pregnant state [6]. Our current aim was to compare the mechanism of TL and PTL initiation using a mouse model of gestation; therefore, only late pregnant (GD15), labouring and PP samples were included in the analysis. Present data show that there was an increase in the expression of multiple cytokine genes commonly associated with inflammation in the uterine muscle (myometrium) during TL. Protein expression analysis, however, revealed that majority of the cytokine proteins were elevated shortly after birth, temporally following changes in cytokine transcripts. FACS analysis was performed to quantitatively assess major immune cell subpopulations infiltrating mouse myometrium before and during TL and PTL. Stereological IHC analysis confirmed the presence of leucocytes in the mouse myometrium before, during and after labour, providing an evidence of the immune cell extravasation into maternal uterine tissue. Importantly, we detected a strong inflammatory response after the onset of term and PTL (during early PP period). Our data support the hypothesis of Mitchell and Taggart that human labour may involve an interweaving of the pro-contractile and pro-inflammatory systems which integrates and amplifies uterine contractile activity and initiates preparedness of the immune system for the critical healing/remodelling that occurs within the uterus immediately following birth [23].

Of note, we found a similar pattern of cytokine expression in TL and RU486-induced PTL. This is expected, as progesterone withdrawal is fundamental to labour initiation [24, 25]. In addition, similarity in the cytokine expression profile between the TL and RU486-induced PTL was observed in the PP period. Interestingly, anti-inflammatory cytokine expression decreased in association with labour at term and RU486-PTL, thus we suggest a switch in the balance between pro- and anti- inflammatory cytokines occurs under these two conditions. LPS-induced (infectious) PTL was associated with more dramatic increase, accelerated induction and broader range of cytokines than that was seen with TL and RU486-PTL. The involvement of a greater number of cytokines and exaggerated levels of cytokine expression was maintained throughout the PP period. There was an observed increase in the anti-inflammatory cytokine Il13 in both models of PTL in the PP period, which may serve to prevent an exacerbated immune response by balancing pro-inflammatory cytokine secretion. Importantly, the chemokine component of cytokine profiles in all three mouse models was similar during the PP period, suggesting that the leucocyte recruitment into the myometrium serves a similar biological function at this time.

As we proposed earlier, the myometrium should be considered as an immune regulatory tissue that play an essential role in the programming of leucocyte infiltration in the pregnant uterus [6]. Multiple experiments performed by our group and others clearly indicate that myometrial SMCs (both human and animal origin) are able to produce multiple cytokines before and during labour *in vivo* and when cultured *in vitro* [6, 26]. We have shown recently that myometrially secreted chemokines are biologically active and can initiate the infiltration of immune cells into the uterus [6, 27]. We proposed that the mechanical stretch exerted physiologically as a result of embryo growth contributes to the chemokine-mediated infiltration of immune cells into the myometrium during TL [6, 26]. This local autocrine secretion of cytokines by mechanically and hormonally primed myometrial SMCs may serve as the first directional signals for peripheral leucocytes to enter the tissue. In agreement with this hypothesis, we found a significant increase in number of myometrial macrophages before the onset of term and in neutrophils–after birth. Surprisingly we could not detect an increase in cytokine proteins in crude myometrial tissue lysates from term labouring mice, but only in myometrial samples collected after delivery. We speculate now that the high local concentration of specific cytokines around the local vasculature responsible for the attraction of monocytes/macrophages into the myometrium may be curtained in these samples as they were extracted from bulk uterine tissue. It is equally plausible that decidua could play a key role in the labour process by coordinating inflammatory events through paracrine crosstalk between the adjacent myometrium and foetal membranes. Decidual leucocytes and/or decidual cells itself may activate the adjacent myometrium through up-regulation of chemokines, resulting in myometrial activation [28]. The cytokine signature and immune cell presence in mouse term and preterm decidua is a focus of our future investigations. We will also examine the timing of decidual activation in relation to myometrial activation and PP uterine involution.

The precise role of specific cytokines in the process of labour initiation remains to be determined. As multiple cytokines can activate the same downstream effectors (such as PGs synthesis and CAP gene expression), the need for such broad chemokines activation is unresolved. We detected a dramatic increase in *Pghs2* expression during TL and both PTL models which led us to speculate that there is an association between pro-inflammatory cytokines influencing *Pghs2* expression independent of the stimulus for labour, systemic or acute. Prostaglandin production is intimately involved in the mechanisms of parturition, with PGE_2_, PGF_2α_ and other eicosanoids acting as potent stimulators of myometrium contractions [29]. The inhibition of Pghs2 was shown to prevent infection-induced PTL in mice [30] and in a non-human primate model [31]. It was also reported that increased levels of Pghs2 in mice were associated with PTB [32] and that this phenotype was rescued by oral administration of celecoxib, a selective Pghs2 inhibitor [33]. Cytokines, particularly IL1b and TNF-α, can prime uterine SMCs for contraction and labour *via* increased PGHS2 expression and enhanced prostaglandin production in the amnion, chorion, decidua and myometrium[12, 34, 35]. In addition, Hirsch *et al*. demonstrated that double knockout mice lacking Il1b and TNF-α receptors had lower myometrial levels of Pghs2 mRNA after *E. coli* administration and delivered a day later than wild-type animals [36]. Pro-inflammatory cytokines may also promote TL and PTL through stimulation of uterine contractility by mechanisms involving induction of CAPs. In our study, the CAPs *Otr* and *Gja1* were also up-regulated during TL and RU486-induced PTL. It has been demonstrated that Il6 significantly increases *Otr* mRNA expression in rat uterine explants [37] and cultured uterine smooth muscle cells [38]. Furthermore, the characteristic late-gestation elevation in uterine expression of mRNA encoding *Otr* and *Ptgs2* was delayed in Il6 null mutant mice [39].

Activation of the CAPs gene expression (Ptgs2, Oxtr and Gja1) during TL is a multifactorial process. We and others reported earlier that two separate, but integrated pathways regulate this process in animals: an endocrine cascade comprising the foetal hypothalamic-pituitary-adrenal-placental axis and a mechanical pathway in which foetal growth imposes tension (stretch) on the uterine wall inducing biochemical and molecular changes within the myometrium [40, 41]. There is a great similarity between TL and RU486-PTL where loss of PR function upon RU486 administration induces changes similar to withdrawal of progesterone occurring during normal TL. However, there was a notable dissimilarity in the pattern of CAP genes expression between two PTL models. Intrauterine infusion of LPS resulted in significantly increased myometrial cytokines (Il1a, Il1b, Il5, Il6, Il9 *etc*.), *Ptgs2* and *Otr* expression levels, but *Gja1* levels did not change during LPS-induced PTL. We suggest that this may be attributed to the difference in the timing of delivery between RU486- and LPS-induced PTL which was more rapid than RU486-induced PTL. We speculate that in contrast with TL, during LPS-induced PTL the huge increase in prostaglandin production could be sufficient to overcome the poorly coordinated myometrial contractions due to the lack of *Gja1*(*Cx43*). In addition, Cx43 is only one member of at least 16 proteins encoded by this family of gap junction genes. It is likely that the expression of other connexin isoforms near term (*i.e. Gjb2/Cx26*) may compensate and/or contribute to the muscle contraction during LPS-PTL.

Our results correspond well with other studies that have investigated molecular markers of labour activation in cervix. Holt *et al*. [42] examined the mechanism of term and preterm cervical ripening in mouse models of TL, infectious PTL and in sterile PTL. They reported that during RU486-induced (sterile) PTL, mouse cervix underwent an acceleration of the processes observed during term ripening, however, they found that different mechanisms regulated premature ripening in an infection-induced PTL. Cytokine levels in the mouse cervix were up-regulated in both RU486- and LPS-induced PTL models, with significantly greater levels of up-regulation during LPS-induced PTL [42]. Studies on mouse myometrium (current) and cervix [42] show different patterns in the expression of immunomodulators in the two mouse PTL models, however, whether or not it involves different mechanisms of labour remains to be determined. In support of different mechanisms operating in TL and LPS-PTL, Gonzalez *et al*. also observed an increase in the expression of genes involved in immunity and inflammation in the mouse cervix of LPS-induced PTL as compared to TL[17]. Importantly, they also reported that the expression of cytokines did not increase in the cervix during TL, but increased during PP involution which corroborate well with our own results and point to the fact that cytokine secretion may facilitate the recruitment of immune cells into the uterus after labour.

It is known that cytokines released by inflamed tissues greatly enhance leucocyte migration by increasing adhesion molecule expression on endothelial cells and also up-regulating the corresponding receptor expression on circulating leucocytes [43]. Typical responses rely on a cascade of events between immune cells from local blood vessels and endothelial cell adhesion molecules finalizing in transmigration of leucocytes across the endothelial layer, a process that takes up to 7 days to accomplish [44, 45]. The resident leucocytes, however, are able to instantly respond to stimuli by up-regulating adhesion molecules and rapidly recruit more leucocytes to the site of inflammation [46]. Our flow cytometry data confirmed by quantitative IHC show that large number of macrophages extravasate into myometrium prior to labour (GD18), and during TL decreasing at PP period. Similar to present results, Mackler *et al*. [47] observed a peak in cervical macrophage numbers at or around GD18 in mice, with a decline to non-pregnant macrophage levels by day one PP. The timing of the macrophage increase in pregnant mouse myometrium before TL suggests that they may contribute to the initiation of labour, however, it is equally possible that the increased myometrial macrophages before TL may be required for the rapid infiltration and function of immune cells (possibly neutrophils, monocytes or T cells) during PP involution process. As suggested by our present cytokine protein expression data, the activation of macrophages by specific myometrial cytokine(s) may also happen after the delivery of the foetus.

We and others show that macrophages infiltrate the myometrium and decidua before and during idiopathic PTL [5, 6, 28]. Macrophages themselves are a rich source of pro-inflammatory cytokines, prostaglandins, proteases and reactive oxygen species (rev in [28]). Hence, they are capable of initiating and amplifying inflammatory responses, activating the decidua, the adjacent myometrium, cervix and foetal membranes, and finally triggering labour. However, the pattern of macrophage and neutrophil infiltration differed in PTL cases as there was no increase in macrophages in contrast with the increase observed during TL. This may be due to the premature and rapid timing of events, which may not allow sufficient time for infiltration of monocytes and their differentiation to macrophages to occur. Resident macrophages, however, are present in the myometrium on GD15 and their rapid activation by multiple cytokines (infection-induced in LPS model or hormonal regulated in RU486 model) may contribute to the process of PTL and the large increases in neutrophil infiltration seen in the LPS group during the PP period.

Macrophage infiltration before term parturition in uterine muscle is followed by neutrophil infiltration after delivery. This is in good agreement with data previously reported by Timmons *et al*., who showed that neutrophil numbers do not increase in the mouse cervix until after birth, suggesting a role for these cells in PP remodelling of the cervix rather than in the initiation of cervical ripening at parturition [48]. Correspondingly, the chemokines that were up-regulated PP (Ccl2, Ccl3 and Cxcl1) are known to be strong chemoattractants for circulating monocytes and neutrophils and could be produced by activated macrophages [10, 49–51]. This suggests that macrophage activation during labour may be critical for the process of uterine involution immediately following birth [52]. In agreement, we demonstrated an increase in the number of peripheral monocytes and neutrophils infiltrating the mouse myometrium during the PP period. We also recorded an increase in PP myometrial expression of Il6 and Il12p40 which can support the differentiation of monocytes to macrophages and/or activation of differentiated macrophages [12, 39, 53]. Importantly, recent studies by Menzies *et al*. reported that mice deficient in Ccl2 receptor CCR2 have normal parturition despite reduced monocytes trafficking into the uterus. This study supports the potential importance of myeloid cells in PP uterine involution rather than initiation of term labour [54].

Previous studies in rat skeletal muscle have indicated that macrophages and neutrophils play a significant role in muscle regeneration [55]. Two distinct waves of macrophage infiltration were observed in the injured tissue. The first wave of phagocytic macrophages degraded the contents of the injured muscle fibres and was followed by a second wave of non-phagocytic macrophages able to release soluble substances that influence proliferation, differentiation, growth, repair and regeneration of muscle cells. We suggest that the process of PP uterine involution is similar to that of the muscle repair. The likely role of neutrophils in smooth muscle repair and remodelling is the oxidative or proteolytic modification of damaged tissue, allowing phagocytosis of debris by macrophages or other types of neutrophil [55]. PP involution is a complex biological process that requires interactions between different cells, including uterine myocytes, fibroblasts and immune cells. These interactions could involve numerous growth factors, hormones, pro-inflammatory cytokines and chemokines [41]. We reported earlier that myometrial proliferation was significantly reduced during late gestation as compared to an earlier stage of pregnancy and reactivated later during the PP stage of myometrial involution [56]. In parallel with the ability to attract leucocytes, chemokines can induce cellular proliferation, differentiation, apoptosis and angiogenesis [11]. We suggest, therefore, that a repair process similar to that observed in injured skeletal muscle may occur during the regeneration of myometrial smooth muscle after labour under the influence of specific chemokines secreted by uterine and immune cells.

In summary, our data demonstrate that the myometrium acts as an immune regulatory tissue to orchestrate the contribution of multiple subsets of immune cells to the process of labour and PP involution. This process appears to be tightly controlled likely through chemokines/cytokines secreted by the smooth muscle cells themselves. These factors control the temporal recruitment of waves of macrophages, monocytes and neutrophils linking the activation of the immune system before and during labour with the process of tissue repair and regeneration after birth. We share the hypothesis of Mitchell and Taggart that labour mechanism might be activated simultaneously with the mechanism of healing/involution process following delivery, and might actually represent an evidence of a biological system strength designed to efficiently orchestrate parturition and uterine involution [23]. Our results highlight the need to further investigate the biological processes that occur in the uterus after delivery of the baby and placenta as failure of uterine contractility during involution may contribute to maternal post-partum haemorrhage and morbidity. The recognition of the importance of the communication between the myometrium and immune system presents new potential targets by which the process of labour can be regulated and complication of labour, both preterm and post-term, might be prevented.

## Supporting information

**Fig. S1** Changes in cytokine protein levels in the mouse myometrium during normal gestation, term labour and post-partum (TL group), LPS-induced PTL and post-partum (LPS PTL group) and RU486-induced PTL and post-partum (RU PTL group). A and B: Cytokines (Il1a, Il3, Il4, Il5, Il9, Il12(p70), Il13, Il17 and Infg) and C. Chemokines Csf3 (Gcsf), Eotaxin and Ccl5 (Rantes) protein expression were detected by multiplex magnetic bead assay. Shown are non-labouring samples (GD15, RU Vehicle or LPS Sham, white bars), term and preterm labouring samples (grey bars) or myometrium samples collected 2–6 hrs post-partum (black bars). Results were expressed as mean±SEM (*n* = 8-10). Significant difference with GD15/RU486 Vehicle/Sham LPS is indicated by * (*P* < 0.05) and ** (*P* < 0.01).

**Fig. S2** FACS analyses of immune cells infiltrating mouse myometrium during TL and PTL. Myometrial suspensions were stained with anti-CD45, anti-Gr1 and anti-Neu 7/4. Live leucocytes were gated on CD45 and further analysed on the Neu7/4 *versus* F4/80 dot plot. Neu7/4^++^ cells were further analysed on the Neu7/4 *versus* Gr1 plot or the Siglec F *versus* Gr1 dot plot. Monocytes were identified as Neu7/4^++^ and Gr1^low^ to Gr1^+/−^ cells. Neutrophils were defined as Neu7/4^+^, Gr1^++^ population. Macrophages were defined as F4/80^++^, Neu7/4^low^ cells. Eosinophils were defined as Gr1^low^, SiglecF^++^ cells. (A) Neutrophils and monocytes infiltrate the myometrium during normal gestation, TL and PP (B) during LPS-induced PTL and RU486-induced PTL. Macrophages are the largest population of immune cells present in pregnant mouse myometrium. They increased in number by GD18 (term) as compared to GD15.5 and were elevated during spontaneous TL.

**Fig. S3.** *In situ* localization of leucocytes in mouse myometrium during TL and PTL. Macrophage (A,C) and neutrophils (B,D) infiltration in the myometrium during normal gestation and TL, during LPS-induced PTL and during RU486-induced PTL. Neutrophils were identified using anti-Neu7/4 antibody and macrophages - using anti-F4/80 antibody. Leucocytes (arrows) stained dark brown in paraffin-embedded tissue sections that were counterstained blue with Haematoxylin. Representative photomicrographs of (A) macrophages (F4/80) and (B) neutrophils (Neu7/4) in the myometrium during gestation and term labour. Immunohistochemical examination was performed on sections of uterus from gestational days 15, 18, during term labour (LAB) and 2–6 hrs post-partum (PP). (C) Macrophage (F4/80) and (D) neutrophil (Neu7/4) infiltration in the myometrium during LPS- and RU486-induced PTL mouse models. Immunohistochemical examination was performed on sections of uterus from gestational days 16 (Sham or RU Vehicle), day 15/16 preterm labouring mice (125 LPS PTL or RU PTL) and 2–4 hrs PP after treatment with either LPS or RU486 (125LPS PP or RU PP). The negative controls were performed on GD15 uterine tissue samples: Control 1 represents the lack of immunostaining after incubation of myometrial tissue with non-specific rat IgG and Control 2 shows staining with anti-rat secondary antibodies in the absence of primary antibody. Magnification = 200x; scale =20 μm.

**Table S1** Changes in cytokine mRNA levels in the mouse myometrium during normal gestation, term labour (TL) and postpartum (PP) in fold change *versus* GD15; LPS-induced preterm labor (LPS-PTL) and postpartum (LPS-PP) in fold change *versus* Sham sample and RU486-induced preterm labor (RU-PTL) and postpartum (RU-PP) in fold change *versus* Vehicle sample.

**Table S2** Changes in cytokine protein levels (pg/ml) in the mouse myometrium during normal gestation (GD15), term labour (TL) and postpartum (PP), RU486-induced preterm labor (RU-PTL), Vehicle-treated GD15 (Vehicle) and postpartum (RU-PP), LPS-induced preterm labor (LPS-PTL), sham operated GD15 (Sham) and postpartum (LPS-PP).
