# Supplementary material for: Myometrial immune cells contribute to term parturition, preterm labour and post-partum involution in mice
Source: J Cell Mol Med. 2012 Dec 4;17(1):90–102. doi: 10.1111/j.1582-4934.2012.01650.x (PMC3823139; doi:10.1111/j.1582-4934.2012.01650.x)
Supplement: Supplementary file 1 [file jcmm0017-0090-SD1.ppt]

## Slide 1
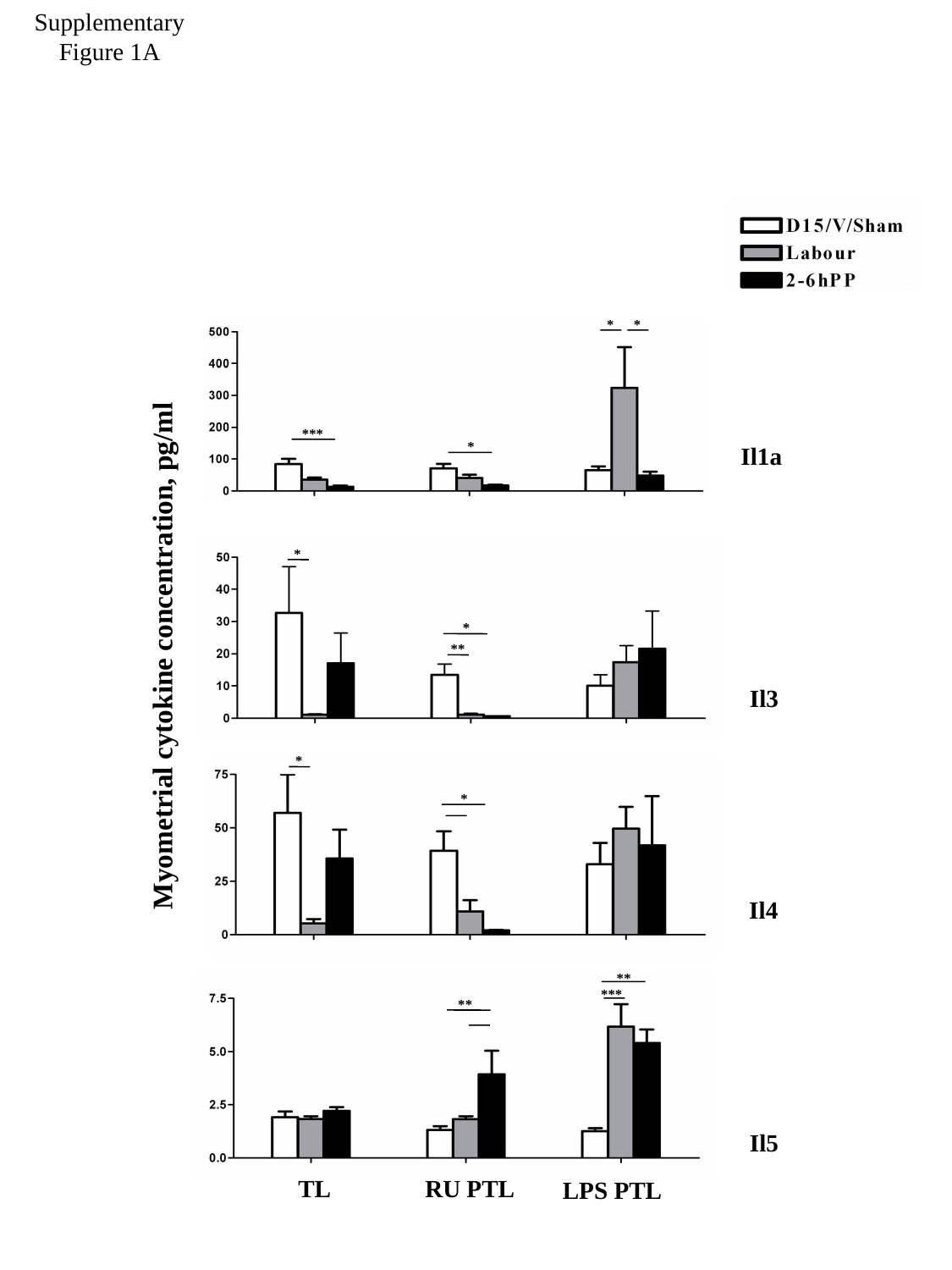

Supplementary Figure 1A
*
*
***
*
Il1a
*
*
*
Myometrial cytokine concentration, pg/ml
**
Il3
*
*
Il4
**
***
**
Il5
TL
RU PTL
LPS PTL

## Slide 2
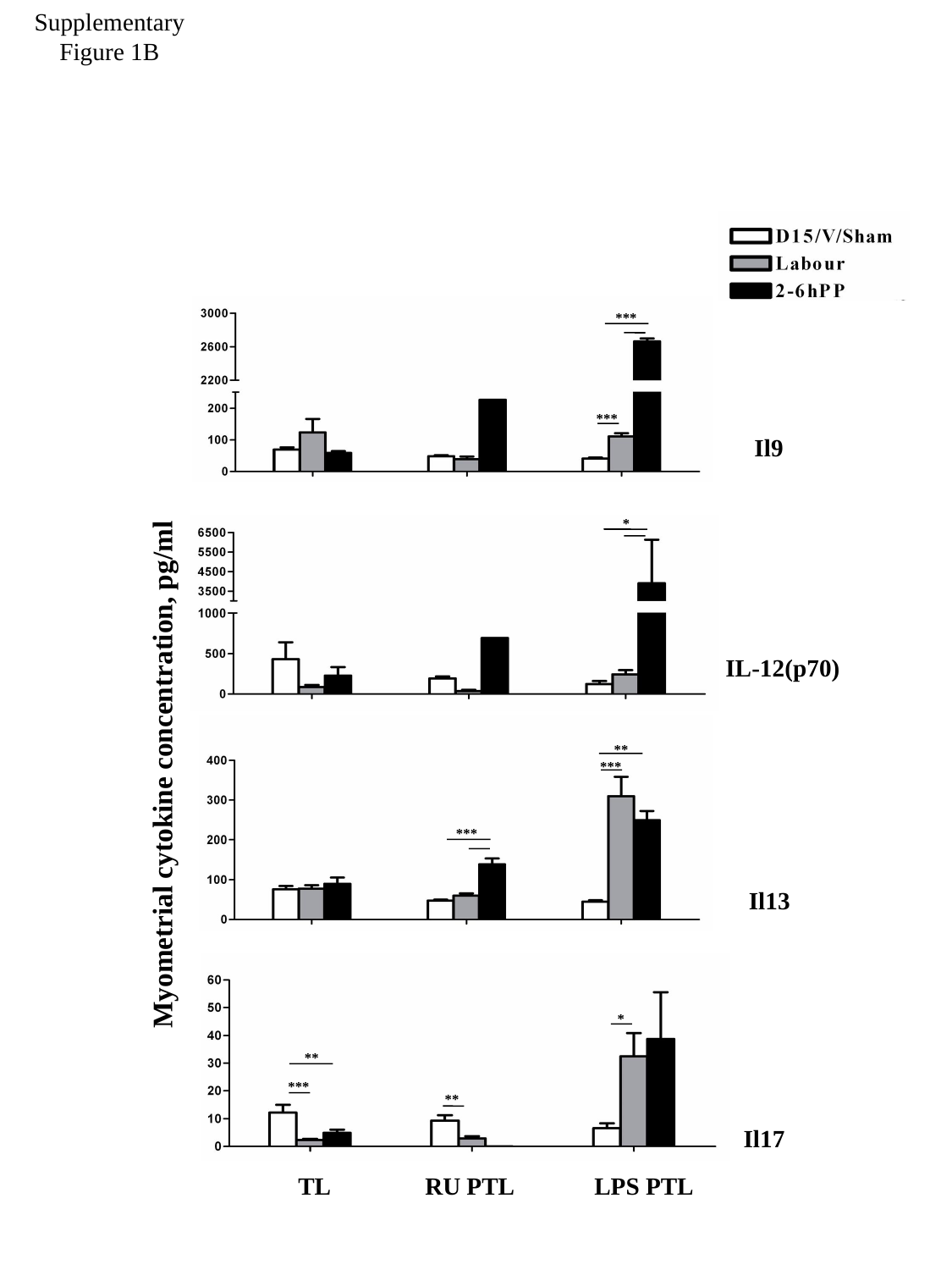

Supplementary Figure 1B
***
***
Il9
*
IL-12(p70)
**
Myometrial cytokine concentration, pg/ml
***
***
Il13
*
**
***
**
Il17
TL
RU PTL
LPS PTL

## Slide 3
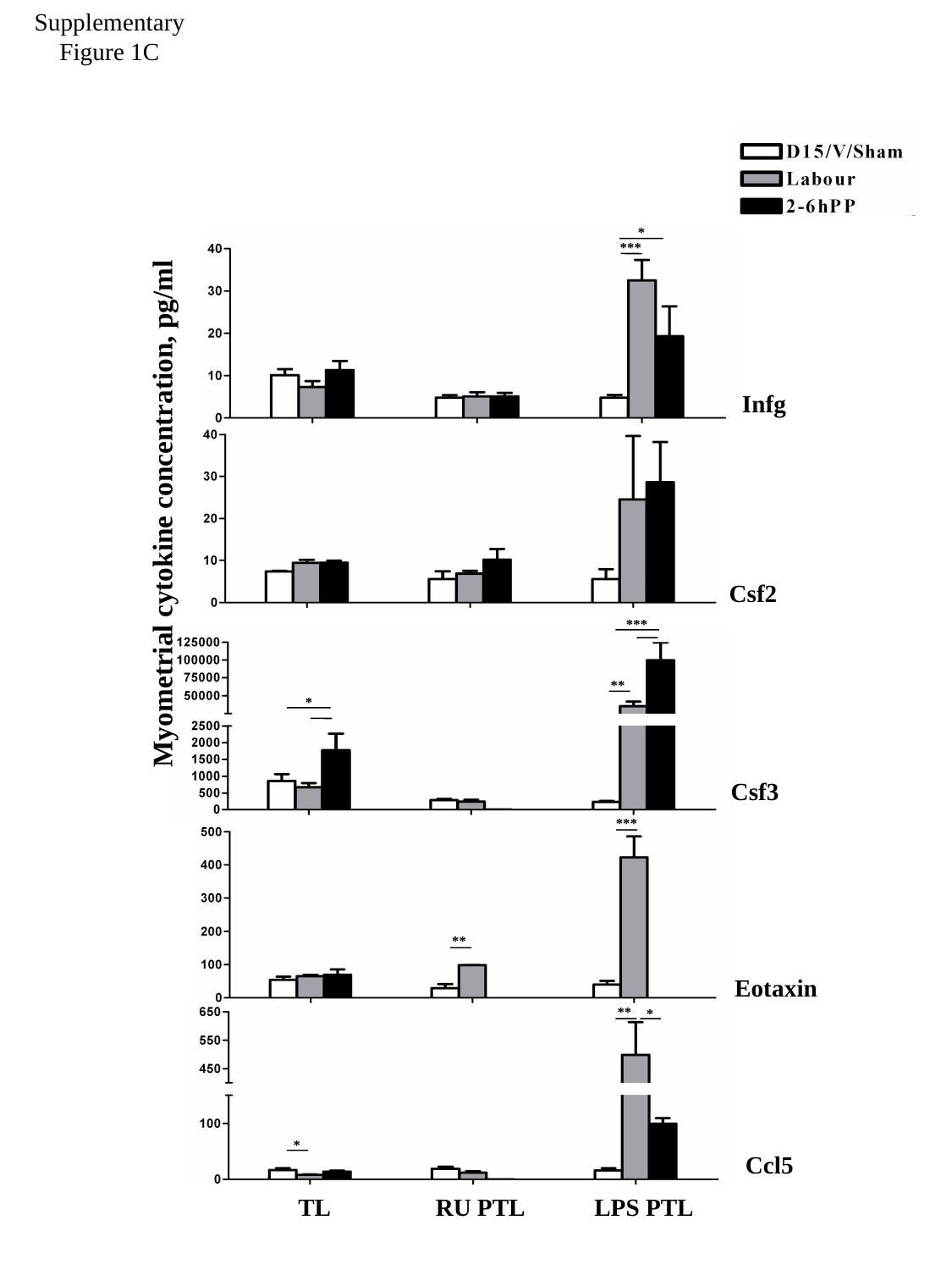

Supplementary Figure 1C
*
***
Infg
Myometrial cytokine concentration, pg/ml
Csf2
***
**
*
Csf3
***
**
Eotaxin
**
*
*
Ccl5
TL
RU PTL
LPS PTL
