# Supplementary material for: Myometrial immune cells contribute to term parturition, preterm labour and post-partum involution in mice
Source: J Cell Mol Med. 2012 Dec 4;17(1):90–102. doi: 10.1111/j.1582-4934.2012.01650.x (PMC3823139; doi:10.1111/j.1582-4934.2012.01650.x)
Supplement: Supplementary file 2 [file jcmm0017-0090-SD2.ppt]

## Slide 1
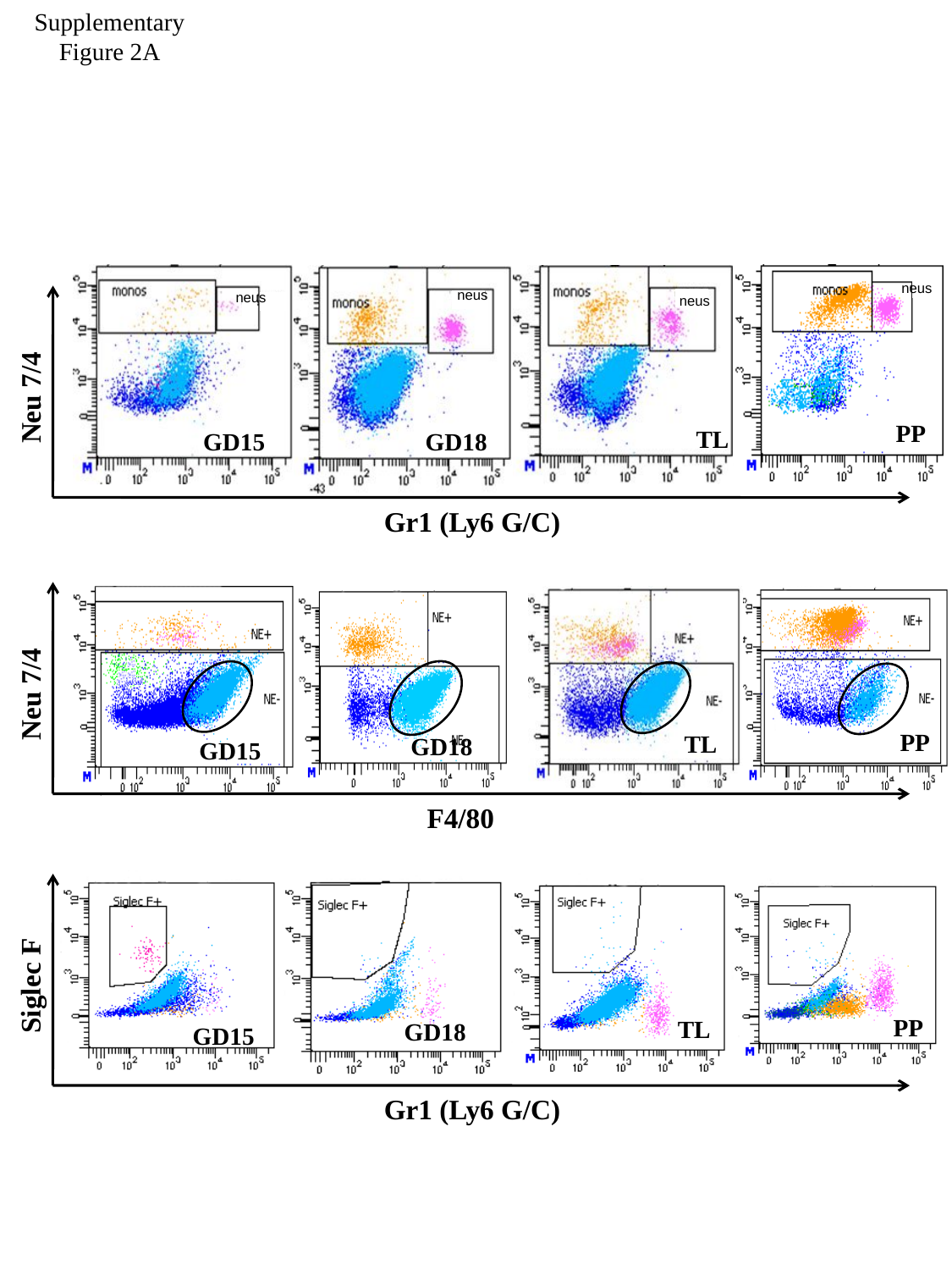

Supplementary Figure 2A
neus
neus
neus
neus
Neu 7/4
PP
TL
GD15
GD18
Gr1 (Ly6 G/C)
Neu 7/4
PP
TL
GD18
GD15
F4/80
Siglec F
PP
TL
GD18
GD15
Gr1 (Ly6 G/C)

## Slide 2
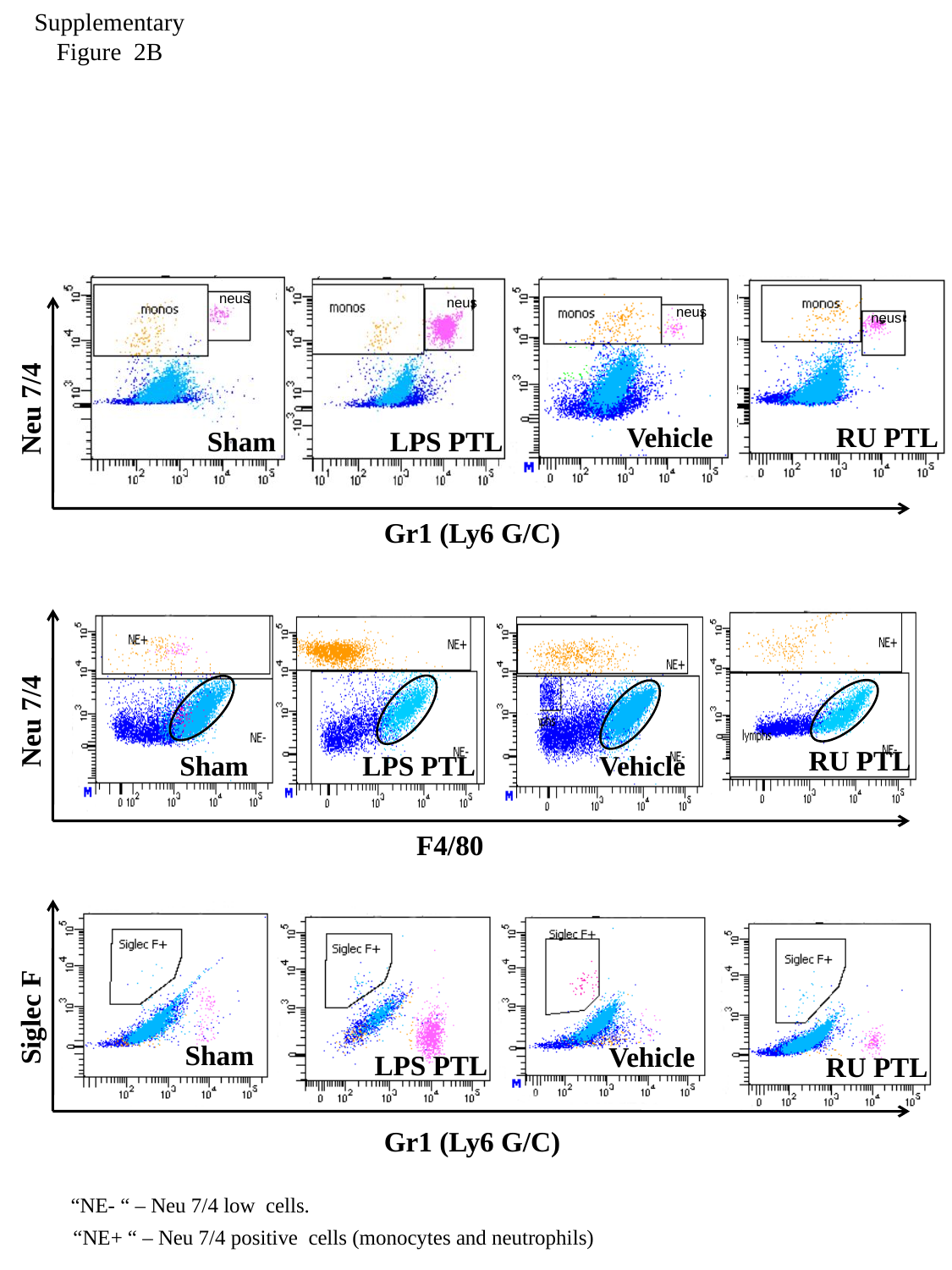

Supplementary Figure 2B
Vehicle
neus
neus
neus
neus
Neu 7/4
RU PTL
Sham
LPS PTL
Gr1 (Ly6 G/C)
Neu 7/4
RU PTL
Sham
LPS PTL
Vehicle
F4/80
Siglec F
Sham
Vehicle
Vehicle
LPS PTL
RU PTL
Gr1 (Ly6 G/C)
“NE- “ – Neu 7/4 low cells.
“NE+ “ – Neu 7/4 positive cells (monocytes and neutrophils)
