# Supplementary material for: Myometrial immune cells contribute to term parturition, preterm labour and post-partum involution in mice
Source: J Cell Mol Med. 2012 Dec 4;17(1):90–102. doi: 10.1111/j.1582-4934.2012.01650.x (PMC3823139; doi:10.1111/j.1582-4934.2012.01650.x)
Supplement: Supplementary file 3 [file jcmm0017-0090-SD3.ppt]

## Slide 1
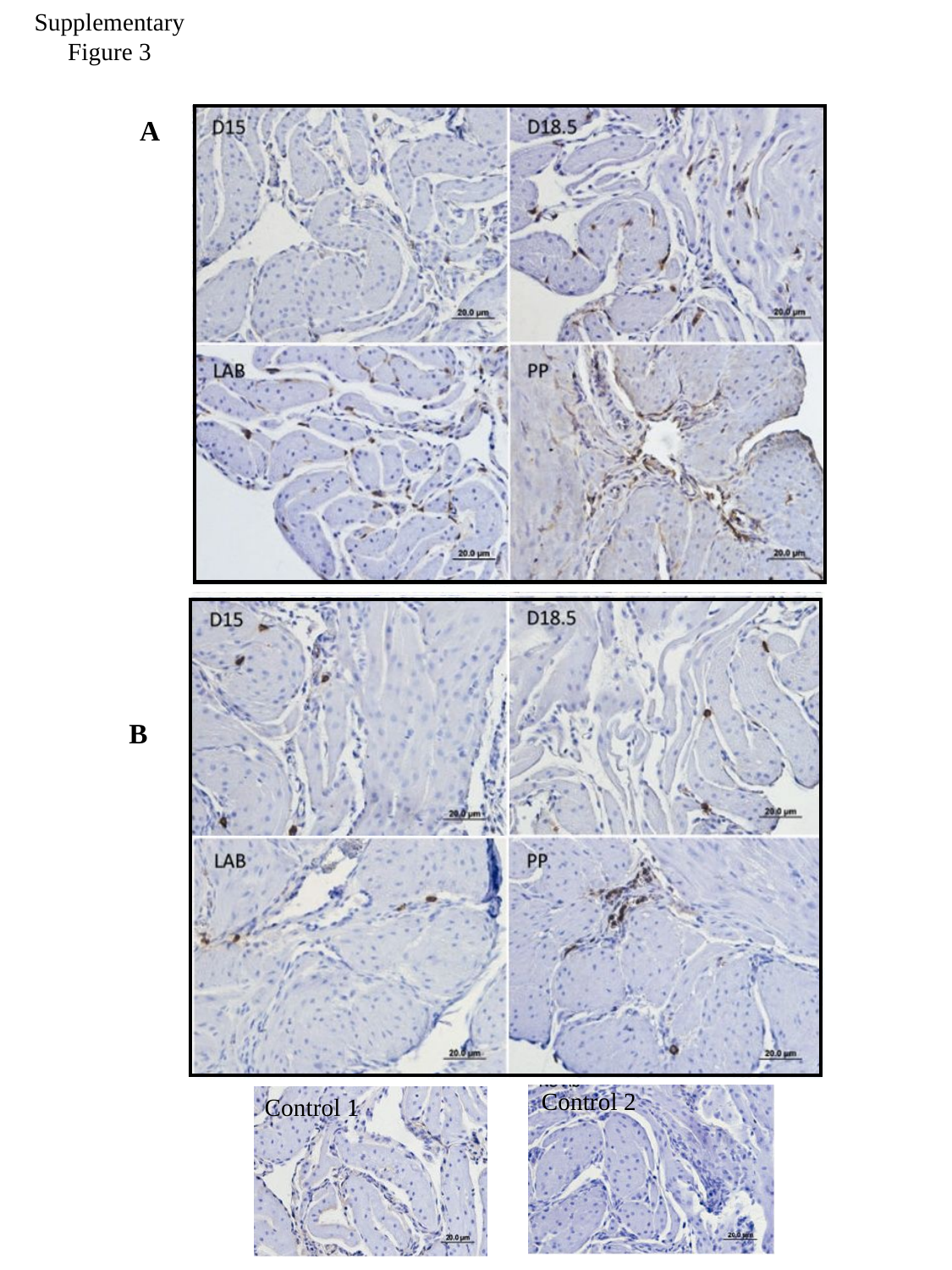

Supplementary Figure 3
A
B
Control 2
Control 1

## Slide 2
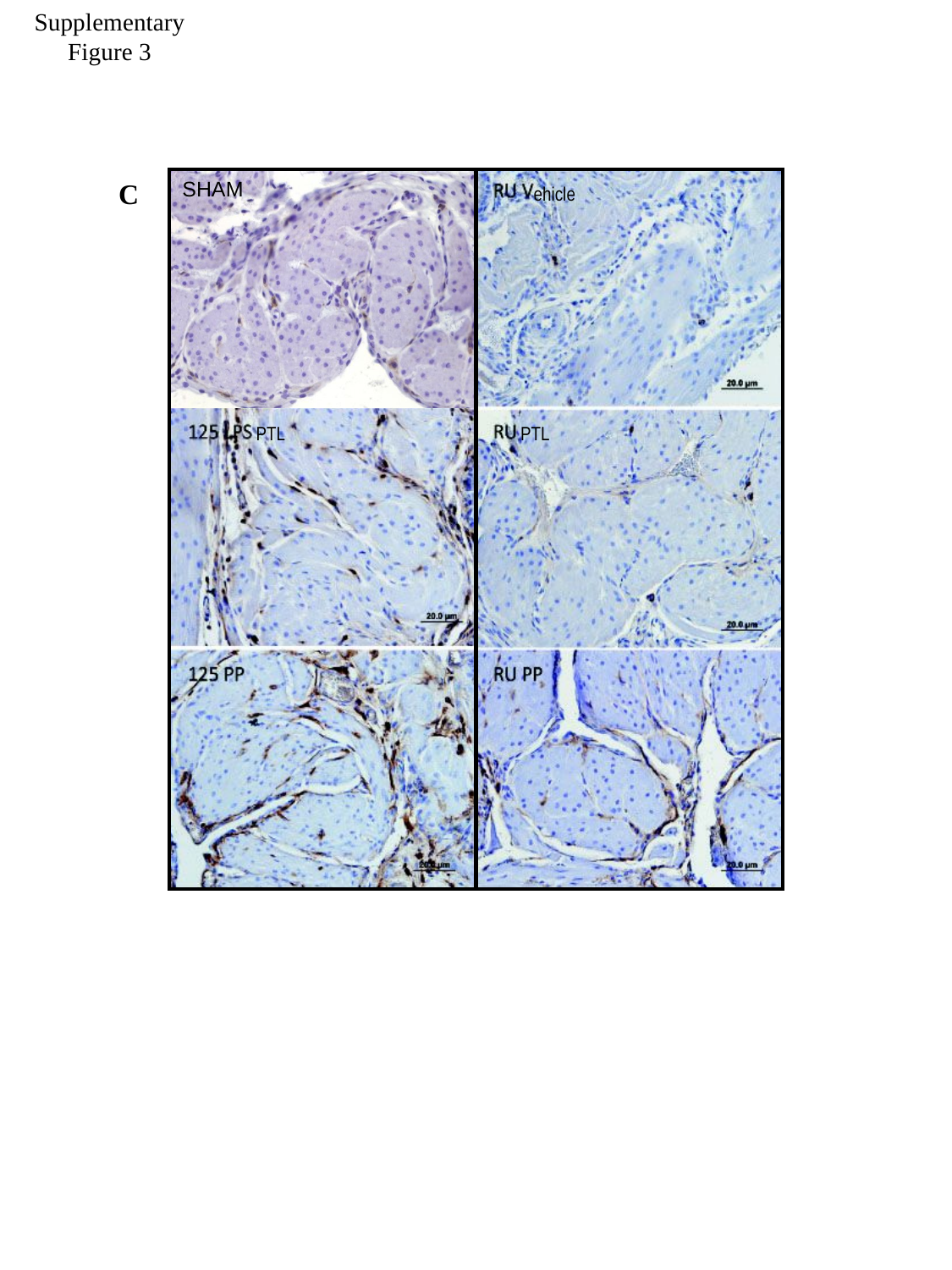

Supplementary Figure 3
C
SHAM
ehicle
PTL
PTL

## Slide 3
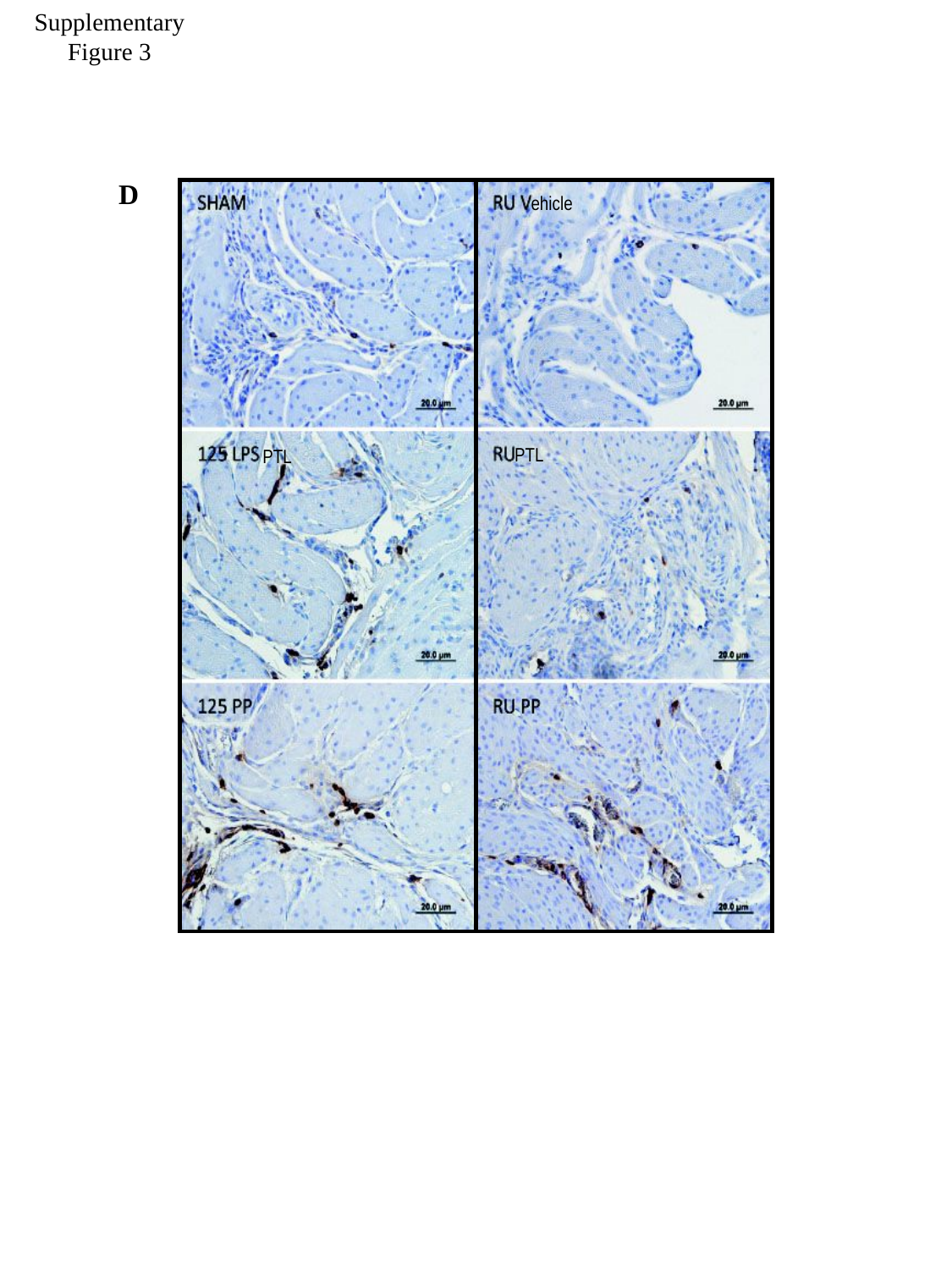

Supplementary Figure 3
D
ehicle
PTL
PTL
