# Supplementary material for: Myometrial immune cells contribute to term parturition, preterm labour and post-partum involution in mice
Source: J Cell Mol Med. 2012 Dec 4;17(1):90–102. doi: 10.1111/j.1582-4934.2012.01650.x (PMC3823139; doi:10.1111/j.1582-4934.2012.01650.x)
Supplement: Supplementary file 4 [file jcmm0017-0090-SD4.docx]

**Table 2.** Changes in cytokine mRNA levels in the mouse myometrium during normal gestation, term labour (TL) and postpartum (PP) in fold change vs GD15; LPS-induced preterm labor (LPS-PTL) and postpartum (LPS-PP) in fold change vs Sham sample and RU486-induced preterm labor (RU-PTL) and postpartum (RU-PP) in fold change vs Vehicle sample.

| ***Cytokines*** | **GD15** | **TL** | **2-6 h PP** | **Vehicle** | **RU-PTL** | **RU-PP** | **Sham** | **LPS-PTL** | **LPS-PP** |
| --- | --- | --- | --- | --- | --- | --- | --- | --- | --- |
| ***Tnfa*** | 1±0.3 | 3.7±1.7* | 3.2±1.9 | 1±0.4 | 2.5±1.2† | 3.2±1.9* | 1±0.1 | 15.9±4.8* | 7.3±1.5 |
| ***Il1b*** | 1±0.3 | 8.6±4.5* | 6.3±2.7* | 1±0.2 | 6.7±5.1* | 5.0±2.7* | 1±0.7 | 28.9±16.6* | 16.6±12.0 |
| ***Il12b*** | 1±0.2 | 20.9±33.0* | 21.9±26.2* | 1±0.5 | 3.4±1.0* | 4.9±2.3* | 1±0.7 | 1.2±1.2 | 1.9±2.6 |
| ***Il6*** | 1±0.4 | 19.4±14.8* | 2.2±1.2* | 1±0.2 | 10.4±6.9* | 4.8±4.1 | 1±0.6 | 143.2±154* | 21.2±13.2 |
| ***Il10*** | 1±0.6 | 1.7±1.0 | 1.1±0.2 | 1±0.4 | 1.0±0.4 | 1.4±0.3 | 1±0.1 | 2.4±1.4 | 3.1±0.3 |
| ***Cxcl1*** | 1±0.4 | 9.2±3.4* | 9.0±5.4 | 1±0.3 | 5.6±3.6 | 4.6±1.7 | 1±0.9 | 90.3±41.3* | 28.3±10.6 |
| ***Cxcl2*** | 1±0.4 | 13.6±11.4* | 16.3±13.7 | 1±0.4 | 3.3±1.8 | 5.0±1.5* | 1±0.8 | 98.3±72.9* | 28.9±13.6* |
| ***Ccl2*** | 1±0.2 | 3.2±2.7 | 2.7±0.8 | 1±0.2 | 2.5±0.7 | 7.6±1.5* | 1±0.3 | 20.8±8.6* | 20.8±2.1* |
| ***Ccl3*** | 1±1.4 | 1.2±0.5 | 13.3±14.8* | 1±0.2 | 3.1±1.1*† | 6.0±2.3* | 1±0.3 | 21.0±9.6* | 8.1±1.3* |
| ***Ccl4*** | 1±1.3 | 1.8±0.6 | 17.5±18.6 | 1±0.5 | 2.9±1.0 | 8.4±3.5* | 1±0.2 | 51.9±30.9* | 8.2±2.1* |
| ***Csf2*** | 1±1.2 | 4.5±4.8 | 39.9±33.4* | 1±0.5 | 2.8±2.0* | 0.1±0 | 1±0.8 | 5.0±2.5*† | 1.1±1.2 |

Results were expressed as mean +/- SD

*- different from GD15/Vehicle/Sham (p<0.05)

†- different from PP (p<0.05)
