# Supplementary material for: Myometrial immune cells contribute to term parturition, preterm labour and post-partum involution in mice
Source: J Cell Mol Med. 2012 Dec 4;17(1):90–102. doi: 10.1111/j.1582-4934.2012.01650.x (PMC3823139; doi:10.1111/j.1582-4934.2012.01650.x)
Supplement: Supplementary file 5 [file jcmm0017-0090-SD5.docx]

**Table 3.** Changes in cytokine protein levels (pg/ml) in the mouse myometrium during normal gestation (GD15), term labour (TL) and postpartum (PP), RU486-induced preterm labor (RU-PTL), Vehicle-treated GD15 (Vehicle) and postpartum (RU-PP), LPS-induced preterm labor (LPS-PTL), sham operated GD15 (Sham) and postpartum (LPS-PP).

| **Cytokines** | **GD15** | **TL** | **2-6 h PP** | **Vehicle** | **RU-PTL** | **RU-PP** | **Sham** | **LPS-PTL** | **LPS-PP** |
| --- | --- | --- | --- | --- | --- | --- | --- | --- | --- |
| **Il1a** | 83.9±47.7 | 45.7±39.4 | 13.7±10.0* | 70.8±40.2 | 39.7±30.0 | 17.8±4.9* | 64.3±34.6 | 324.1±360*† | 47.9±23.8 |
| **Il1b** | 22.2±6.6 | 18.1±6.8† | 44.1±19.3 | 14.9±5.8 | 21.7±11.2 | 16.4±5.9 | 14.6±3.3 | 432.3±40.9* | 100.3±58.0 |
| **Il3** | 32.7±40.4 | 1.1±1.0* | 17.1±26.3 | 13.5±9.6† | 2.6±4.3* | 2.4±3.5 | 10.1±9.6 | 17.4±14.3 | 21.5±23.5 |
| **Il4** | 57.1±50.6 | 5.3±6.3* | 35.7±35.6 | 39.3±25.7† | 10.9±14.9* | 2.0±0.4 | 33.0±28.3 | 49.7±28.8 | 41.9±45.9 |
| **Il5** | 1.9±0.8 | 1,8 ±0.9 | 2.2±0.5 | 1.3±0.5† | 2.1±70.9 | 3.9±2.2 | 1.6±0.9 | 6.2±2.8* | 5.4±1.2* |
| **Il6** | 4.3±5.8 | 11.8±16.3†* | 16.7±20.8* | 0.7±0 | 9.0±5.6* | 18.8±18.4* | 1.1±0.6 | 252.8±331.2* | 288.0±232.7* |
| **Il9** | 69.4±17.0 | 124.1 ±94.8 | 58.9±14.6 | 48.5±8.2 | 39.2±17.0 | 227±0 | 41.2±7.8 | 111.0±25.7*† | 2664.7±46.4* |
| **Il10** | 98.2±81.0 | 11.2±7.1* | 42.2±34.4 | 99.3±56.6 | 23.1±30.3* | 20.1±28.8* | 77.6±56.8 | 142.3±78.4 | 121.7±119.4 |
| **Il12 (p40)** | 83.3±33.7 | 135.4±147.1 | 252.2±185.2* | 75.3±43.9 | 149.5±131* | 662.6±556.7* | 59.9±39.9 | 204.6±70.0* | 198.9±113.4* |
| **Il12 (p70)** | 432.9±591.9 | 86.7 ±81.9 | 229.0±296.2 | 194.3±62.1 | 44.4±37.6 | 696.2±0 | 124.8±98.8 | 244.9±141.0*† | 3906.8±4453.2* |
| **Il13** | 76.3±23.4 | 88.2 ±41.0 | 90.3±43.8 | 47.4±7.9 | 69.0±28.6† | 139.2±29.4* | 55.4±30.7 | 309.9±137.1* | 249.1±47.2* |
| **Il17** | 12.3±7.6 | 2.4 ±1.1* | 4.9±3.2* | 9.4±5.6 | 2.9±2.3* | - | 6.6±4.9 | 32.5±24.0* | 38.8±33.6 |
| **Tnfa** | 160.3±133.4 | 32.6±14.5* | 68.2±49.9 | 118.8±71.1 | 39.9±30.0* | 43.8±26.0* | 96.4±67.4 | 173.4±82.7 | 392.6±402.4* |
| **Itfg** | 10.1±4.1 | 7.3 ±4.4 | 11.3±6.1 | 4.8±1.6 | 5.1±2.5 | 5.1±1.7 | 4.8±1.9 | 32.5±13.7* | 19.3±14.1* |

| **Chemokines** | **GD15** | **TL** | **2-6 h PP** | **Vehicle** | **RU-PTL** | **RU-PP** | **Sham** | **LPS-PTL** | **LPS-PP** |
| --- | --- | --- | --- | --- | --- | --- | --- | --- | --- |
| **Cxcl1** | 47.6±33.0 | 66.0±47.2 | 137.1±77.5* | 18.9±6.2 | 48.0±32.7 | 105.9±38.8* | 17.5±6.7 | 1413±440* | 1244±351* |
| **Ccl2** | 107.4±39.3 | 205.7±69.3* | 750.4±381.5* | 60.8±23.3 | 149.1±78.2* | 693.8±409.4* | 62.6±21.0 | 3569±2115* | 3414±1798* |
| **Ccl3** | 16.3±11.4 | 14.3±5.9 | 38.8±16.9* | 16.1±11.3 | 21.5±7.7 | 89.8±64.8* | 11.9±9.2 | 249.1±116.8* | 170.0±55.8* |
| **Ccl4** | 80.3±66.9 | 14.1±7.7* | 43.8±34.2 | 84.8±54.5 | 27.5±28.7 | 25.8±16.5 | 66.8±51.8 | 163.0±71.2* | 97.9±87.6 |
| **Ccl5** | 16.7±9.6 | 7.9 ±3.3* | 13.5±6.2 | 18.8±11.9 | 10.9±7.8 | - | 16.2±10.3 | 498.4±325.5*† | 171.2±143.2* |
| **Csf2** | 7.3±0.2 | 9.4 ±1.9 | 9.4±0.7 | 5.6±2.5 | 6.9±1.3 | 9.3±4.0 | 5.6±3.2 | 24.6±21.3 | 28.6±19.2 |
| **Csf3** | 861.1±575.0 | 671.9±374.4 | 2541±2488* | 283.9±240.7 | 104.7±147.8 | - | 233.1±73.5 | 35297±18197*† | 99716±43752* |
| **Eotaxin** | 53.8±24.9 | 45.7±33.9 | 69.6±39.3 | 34.6±31.3 | 91.5±14.4* | - | 39.7±25.5 | 422.7±154.8* | - |

Results were expressed as mean +/- SD;

*- different from GD15/Vehicle/Sham (p<0.05);

†- different from PP (p<0.05).
